# Validation and cross-cultural adaptation of the COMPASS-31 in Croatian and Serbian patients with multiple sclerosis

**DOI:** 10.3325/cmj.2017.58.

**Published:** 2017-10

**Authors:** Jelena Drulović, Anđela Gavrilović, Luka Crnošija, Darija Kisić-Tepavčević, Magdalena Krbot Skorić, Jovana Ivanović, Ivan Adamec, Irena Dujmović, Anamari Junaković, Gorica Marić, Vanja Martinović, Tatjana Pekmezović, Mario Habek

**Affiliations:** 1 Clinic of Neurology, CCS, Faculty of Medicine, University of Belgrade, Belgrade, Serbia; 2 Clinical Hospital Center Zvezdara, Belgrade, Serbia; 3 School of Medicine, University of Zagreb, Zagreb, Croatia; 4 Institute of Epidemiology, Faculty of Medicine, University of Belgrade, Serbia; 5 University Hospital Center Zagreb, Department of Neurology, Referral Center for Autonomic Nervous System Disorders, Zagreb, Croatia

## Abstract

**Aim:**

To validate and cross-culturally adapt Croatian and Serbian versions of composite autonomic symptom score-31 (COMPASS-31) for the detection of dysautonomia in patients with multiple sclerosis (MS).

**Methods:**

A total of 179 patients, 67 with clinically isolated syndrome (CIS) and 112 with MS, completed the COMPASS-31 at two MS centers in Zagreb and Belgrade between April 1 and October 31, 2016. Demographic and clinical data including age, gender, MS phenotypes, and the Expanded Disability Status Scale (EDSS) score were collected.

**Results:**

The Cronbach’s alpha coefficient of COMPASS-31 total score was 0.844 for the Croatian MS sample and 0.779 for the Serbian MS sample. A joint analysis yielded Cronbach’s alpha coefficients ranging from 0.394 to 0.796, with values in four domains higher than 0.700. In Croatian and Serbian samples and the total study sample, the Cronbach’s alpha coefficient of COMPASS-31 was 0.785. Reproducibility measured by intra-class correlation coefficient (ICC) was acceptable (ICC = 0.795). With regard to the clinical validity, significant correlation was found between EDSS and the COMPASS-31 total score (*P* < 0.001). Furthermore, significant differences between MS phenotypes were detected for bladder and gastrointestinal domains and for the COMPASS-31 total score (*P* < 0.001, *P* = 0.005, and *P* = 0.027, respectively). Finally, significant differences between MS phenotypes in patients with score >0, which implies the existence of at least one of the symptoms investigated in each domain, were detected for secretomotor and bladder domains (*P* = 0.015 and *P* < 0.001, respectively).

**Conclusion:**

COMPASS-31 represents a valid and acceptable self-assessment instrument for the detection of dysautonomia in MS patients.

In recent years, a high percentage of autonomic nervous system (ANS) involvement in neurological diseases such as multiple sclerosis (MS) has been reported (1). ANS research usually consists of the investigation of patient-reported symptoms using different questionnaires and assessment of the ANS function in the laboratory. The latter shows limited reproducibility because the data are obtained with numerous autonomic function tests, which are indirect, relatively imprecise, and fraught with difficulties. A recently published meta-analysis showed that the proportion of MS patients diagnosed with cardiovascular dysautonomia varies largely depending on the number of tests used (2). The best way to overcome this problem is to use a standardized battery of validated and reproducible tests that can be interpreted in the form of a score. This enables detection, staging, and follow-up of dysautonomia in different neurological and non-neurological disorders. The Composite Autonomic Scoring Scale (CASS) is such a test. It has been successfully used in patients with multiple system atrophy, Parkinson disease, and autonomic neuropathy (3). So far, CASS has only been evaluated in patients with clinically isolated syndrome (CIS) suggestive of MS in whom it can detect dysautonomia, which is restricted to sympathetic nervous system involvement (4).

All above-mentioned problems are even more emphasized when symptoms of ANS dysfunction are investigated. Even patients with severe sympathetic dysfunction, defined as orthostatic hypotension with a decrease in systolic blood pressure greater than 60 mm Hg from baseline during a head-up tilt table test, can be completely asymptomatic during the head-up tilt table test in up to one-third of the cases (5). Furthermore, in conditions such as fibromyalgia, a significant discrepancy between ANS symptoms and objective assessments of ANS dysfunction have been found (6). Therefore, a composite autonomic symptom score-31 (COMPASS-31) was developed and found to be suitable for a widespread use in research and practice (7). COMPASS-31 consists of 31 items quantifying 6 autonomic domains as follows: orthostatic intolerance, vasomotor, secretomotor, gastrointestinal, bladder, and pupillomotor. Although there have been no standardized questionnaires on autonomic dysfunction specifically validated for use in MS patients, COMPASS-31 has been used recently for the evaluation of autonomic dysfunction in MS and confirmed that it can detect ANS dysfunction in MS. This finding implied that autonomic symptoms are present early in the disease and in patients with low disability (8).

The aim of the present study was to validate and cross-culturally adapt Croatian and Serbian versions of COMPASS-31 for the detection of dysautonomia in patients with MS. Additionally, we examined whether COMPASS-31 and its domains produce scores that differ across various MS phenotypes, including CIS.

## PATIENTS AND METHODS

### Patients

A total of 179 subjects, 67 patients with CIS and 112 with MS who received regular follow-up care at the Clinic of Neurology, Clinical Center of Serbia, Belgrade, Serbia and Department of Neurology, University Hospital Center Zagreb, Croatia, between April 1, 2016 and October 31, 2016 were asked to complete the COMPASS-31 questionnaire. The diagnosis of CIS was made if a patient had acute or subacute development of neurological symptoms and/or signs lasting longer than 48 hours in the absence of fever or infection, and with at least one demyelinating lesion larger than 3 mm on the brain and/or spinal cord MRI (9). The diagnosis of MS was made according to the Revised McDonald Criteria 2010 (10). The majority of MS patients from Zagreb, Croatia, belonged to the cohort of patients with CIS in whom special attention was paid to the investigation of ANS dysfunction in the Referral Center for Autonomic Nervous System Disorders.

Data on age, gender, clinical course, and the Expanded Disability Status Scale (EDSS) score (11) were collected by neurologists after the physical examination of each patient.

### COMPASS-31

The COMPASS-31 was translated from English to Serbian and from English to Croatian (12). All translated questionnaire items were first discussed by two independent translators for both languages and then back-translated to English by bilingual translators who were blinded to the original questionnaire. Subsequently, translators, MS specialists, and researchers discussed controversial items to generate the versions of the COMPASS-31 that would be the most appropriate for the cultural environments in Serbia and Croatia for testing MS patients. To check the understanding and interpretation of the translated items by Serbian and Croatian populations, the Serbian and Croatian versions of the questionnaire were tested on five MS patients in each center. The results of these tests were discussed by the same groups of specialists. Patients’ suggestions regarding the questionnaire were also taken into consideration. This stage led to the final, culturally adapted Serbian and Croatian versions of the COMPASS-31.

The COMPASS-31 was scored according to the original recommendations (7). The total score ranges from 0 to 100, with higher values representing more severe symptoms. After expert revisions of the original version, questions and domains were further reduced and a total of 31 questions in 6 domains remained. In our study, the COMPASS-31 was completed by MS patients in the presence of a physician who could provide assistance, if needed.

To assess reproducibility, the same two groups of patients with MS completed the COMPASS-31 again after two weeks.

### Statistical analysis

Descriptive statistical analysis of all variables was performed. Normality of distribution was assessed by Kolmogorov-Smirnov test. For comparison of normally distributed variables, Student’s *t*-test was used, while χ^2^ test was performed for comparison of categorical variables. In case of non-normal distribution, Mann-Whitney U test was used.

Internal consistency of the Serbian and Croatian versions of the COMPASS-31 was evaluated using Cronbach’s alpha coefficient (13), which ranged from 0 to 1. Test-retest reliability of scores was evaluated by calculation of the test/retest correlation (intra-class correlation coefficient, ICC).

Clinical validity was assessed by correlating the mean COMPASS-31 total and domain scores with EDSS score. Spearman’s correlation coefficient was used to determine the relationship between the COMPASS-31 scores and the EDSS score. Differences between groups were assessed by ANOVA and *post hoc* Tukey’s test for parametric variables, and by Kruskal-Wallis and Mann-Whitney *U* test for non-parametric variables.

Receiver operating characteristic (ROC) curve analysis was used to determine the cut-off value of COMPASS-31 total score for the identification of MS patients with and those without the investigated symptoms. In this ROC analysis, the COMPASS-31 total and domain scores were dependent variables and the status of MS patient (presence or absence of autonomic dysfunction) was the independent variable. Separate ROC analyses were performed for each domain of the COMPASS-31.

Significance level was set at *P* < 0.05. Data analysis was performed using SPSS 17.0 (SPSS Inc., Chicago, IL, USA).

## RESULTS

### Translation and validation of the questionnaire

All 179 patients were able to read and comprehend the questionnaire. Most found the questionnaire acceptable, clear, and understandable. None of the items were found to be disagreeable. Median time to complete the questionnaire was 8 minutes (range, 4-31 minutes). Twenty-one (11.7%) patients needed help, 11 (6.1%) patients required help with reading because of lower visual acuity, and 10 (5.6%) patients required writing assistance.

Based on the differences in the frequency of MS phenotypes, there was a difference in all patient characteristics except for gender (Table 1).

**Table 1:** Demographic and clinical characteristics of patients with multiple sclerosis (MS) in Serbia and Croatia*

		No.(%) of MS patients	
Variable	Serbia (n = 102)	Croatia (n = 77)	total (N = 179)
Gender			
men	35 (34.3)	29 (37.7)	64 (35.8)
women	67 (65.7)	48 (62.3)	115 (64.2)
Age (years; mean±SD)	42.2 ± 12.8	33.3 ± 9.4	38.4 ± 12.3
range	19-75	19-57	19-75
EDSS (score; mean±SD)	3.8 ± 2.7	1.9 ± 1.4	2.8 ± 2.1
range	0.0-8.0	0.0-6.5	(0.0-8.0)
MS phenotype			
CIS	11 (10.8)	56 (72.7)	67 (37.4)
RRMS	58 (56.9)	19 (24.7)	77 (43.0)
SPMS	20 (19.6)	1 (1.3)	21 (11.7)
PPMS	13 (12.7)	1 (1.3)	14 (7.8)

*Abbreviations: SD – standard deviation, EDSS – Expanded Disability Status Scale, CIS – clinically isolated syndrome, RRMS – relapsing remitting multiple sclerosis, SPMS – secondary progressive multiple sclerosis, PPMS – primary progressive multiple sclerosis.

The Cronbach’s alpha coefficient of COMPASS-31 domains in Serbian MS sample ranged from 0.409 to 0.820, with three domains reaching a value above 0.700 (Table 2). In the Croatian MS sample, Cronbach’s alpha coefficient of COMPASS-31 domains ranged from 0.412 to 0.851, with four domains reaching a value above 0.700. In the joint analysis, Cronbach’s alpha coefficients ranged from 0.394 to 0.796, with values in four domains being higher than 0.700. Finally, in both Serbian and Croatian sample and the total group the Cronbach’s alpha coefficient of COMPASS-31 was higher than 0.700. Reproducibility measured by ICC was acceptable (ICC = 0.795).

**Table 2:** Descriptive characteristics and internal reliability of composite autonomic symptom score-31 (COMPASS-31) in Serbian and Croatian patients with multiple sclerosis (MS)

	COMPASS-31 domains	
Serbian MS patients	median (interquartile range)	Cronbach α
orthostatic intolerance	0 (0)	0.641
vasomotor	0 (0)	0.409
secretomotor	0 (4.3)	0.662
gastrointestinal	4.5 (6.5)	0.798
bladder	1.7 (3.3)	0.705
pupillomotor	1.0 (1.7)	0.820
total score	13.9 (20.0)	0.779
Croatian MS patients		
orthostatic intolerance	0 (12.0)	0.541
vasomotor	0 (0.8)	0.412
secretomotor	0 (2.1)	0.826
gastrointestinal	2.7 (5.4)	0.778
bladder	0 (1.7)	0.851
pupillomotor	1.0 (1.0)	0.730
total score	9.5 (18.0)	0.844
Total MS patients		
orthostatic intolerance	0 (12.0)	0.610
vasomotor	0 (0.8)	0.394
secretomotor	0 (4.3)	0.725
gastrointestinal	3.5 (6.2)	0.785
bladder	0 (3.3)	0.764
pupillomotor	1.0 (1.7)	0.796
total score	12.2 (19.4)	0.785

### Clinical validity

Statistically significant correlation (*P* < 0.001) was found between EDSS score and the COMPASS-31 total score (Table 3). Additionally, scores in gastrointestinal and bladder domains showed a strong correlation with the EDSS (ρ = 0.333; *P* < 0.001 for gastrointestinal domain, and ρ = 0.563; *P* < 0.001 for bladder domain). One other factor significantly related to the EDSS was secretomotor domain (ρ = 0.179; *P* = 0.019). Orthostatic intolerance, vasomotor and pupillomotor domains were not related to the EDSS.

**Table 3:** Clinical validity of composite autonomic symptom score-31 (COMPASS-31) in Serbian and Croatian patients with multiple sclerosis (MS)

COMPASS-31 domains	Expanded Disability Status Scale	
	Correlation coefficient (ρ)	*P*
orthostatic intolerance	0.079	0.303
vasomotor	0.073	0.341
secretomotor	0.179	0.019
gastrointestinal	0.333	<0.001
bladder	0.563	<0.001
pupillomotor	0.095	0.214
Total score	0.266	<0.001

The mean COMPASS-31 domain scores in four different MS phenotypes (CIS, relapsing-remitting MS, secondary progressive MS, and primary progressive MS) showed significant differences between MS phenotypes in bladder and gastrointestinal domains and total score (Table 4). Statistically significant differences in the proportion of participants with score >0, which implied the presence of at least one of the symptoms investigated in each domain, between MS phenotypes were detected in secretomotor and bladder domains (Table 5).

**Table 4:** Descriptive characteristics of composite autonomic symptom score-31 (COMPASS-31) domains according to the multiple sclerosis (MS) phenotype*

COMPASS-31 domains	MS phenotype (mean±SD)				F	*P*
	CIS (n = 67)	RRMS (n = 77)	SPMS (n = 21)	PPMS (n = 14)		
orthostatic intolerance	5.9 ± 8.0	6.4 ± 8.8	7.4 ± 12.7	8.2 ± 10.5	0.340	0.796
vasomotor	0.3 ± 0.7	0.4 ± 0.8	0.3 ± 0.8	0.6 ± 2.7	0.744	0.527
secretomotor	1.4 ± 2.7	2.2 ± 3.3	2.1 ± 3.0	3.5 ± 2.9	2.143	0.097
gastrointestinal	3.5 ± 3.0	4.4 ± 4.2	6.1 ± 3.9^†^	6.7 ± 3.8^†^	4.499	0.005
bladder	0.8 ± 1.7	1.5 ± 2.0	3.1 ± 2.1^†‡^	4.9 ± 3.3^†‡^	18.890	<0.001
pupillomotor	1.3 ± 1.0	1.3 ± 0.9	1.5 ± 0.9	1.0 ± 0.9	0.463	0.708
Total score	13.2 ± 12.9	16.3 ± 15.6	20.5 ± 18.9	25.2 ± 14.6^†^	3.133	0.027

*Abbreviations: SD – standard deviation, CIS – clinically isolated syndrome, RRMS – relapsing remitting multiple sclerosis, SPMS – secondary progressive multiple sclerosis, PPMS – primary progressive multiple sclerosis.

†Tukey’s *post hoc* test, *P* < 0.01, in comparison with CIS.

‡Tukey’s *post hoc* test, *P* < 0.01 in comparison with relapsing remitting MS.

**Table 5:** Proportion of patients with multiple sclerosis (MS) with a score >0 in each domain of the composite autonomic symptom score-31 (COMPASS-31) according to the MS phenotype

COMPASS-31 domains	No. (%) of patients with MS phenotype				*P^†^*
	CIS	RRMS	SPMS	PPMS	
Orthostatic intolerance	28 (41.8)	31 (40.3)	6 (28.6)	6 (42.9)	0.736
Vasomotor	16 (28.6)	20 (26.0)	4 (19.0)	7 (50.0)	0.186
Secretomotor	18 (26.9)	31 (40.3)	9 (42.9)	10 (71.4)^‡^	0.015
Gastrointestinal	56 (83.6)	63 (81.8)	20 (95.2)	12 (85.7)	0.513
Bladder	19 (28.4)	36 (46.8)	18 (85.7)^‡^	12 (85.7)^‡^	<0.001
Pupillomotor	54 (80.6)	66 (85.7)	13 (61.9)	9 (64.3)	0.051

*Abbreviations: CIS – clinically isolated syndrome, RRMS – relapsing remitting multiple sclerosis, SPMS – secondary progressive multiple sclerosis, PPMS – primary progressive multiple sclerosis.

†Independent-samples Kruskal-Wallis test.

‡Mann-Whitney test, *P* < 0.01, in comparison with CIS.

**Figure 1:**
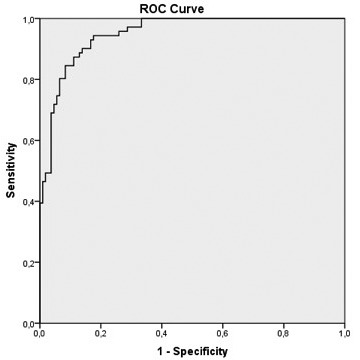
The ROC curve indicating effectiveness of composite autonomic symptom score-31 (COMPASS-31) total score in detection of patients with multiple sclerosis (MS) with the orthostatic intolerance domain score greater than 0. Orthostatic intolerance domain. The area under the curve was 0.950. The optimal cut-off value for COMPASS-31 total score was 13.6, sensitivity was 0.944, and specificity was 0.824. The mean cut-off value for COMPASS-31 total score was 16.3, sensitivity was 0.845, and specificity was 0.898.

The ROC analysis was performed for each domain of the total sample (Figure 1). The highest sensitivity and specificity was detected for the orthostatic intolerance domain. According to the ROC curve for the orthostatic intolerance domain, 95% of the total area was under curve (Figure 1). To differentiate between patients with the orthostatic intolerance score greater than 0 and those with score 0, ie, those with and without autonomic dysfunction, respectively, the optimal cut-off value for the orthostatic intolerance domain score was 13.6. The Serbian and Croatian versions of COMPASS-31 for MS patients who were experiencing orthostatic intolerance symptoms before testing, the sensitivity was 94.4% and the specificity was 82.4% (Figure 1). The ROC curves for the remaining domains are provided as supplementary material (Supplementary Material).

## DISCUSSION

The results of our study showed that Croatian and Serbian versions of COMPASS-31 were valid tools for quantitative assessment of the ANS in MS patients. Furthermore, COMPASS-31 together with some of its domains significantly correlated with the overall disability of MS patients as measured by EDSS. As COMPASS-31 was successfully used in the assessment of various neurological disorders (14,15), our study further expanded its use for the detection of dysautonomia in persons with MS.

We computed the scale reliability, presuming that adequate reliability coefficients in our populations indicated that the scales measured by the COMPASS-31 were appropriately assessed. We found acceptable internal consistency and reliability of the translated versions of the questionnaire. Sletten et al (7) previously demonstrated Cronbach’s alpha coefficient of 0.700 and more for the original US English version and considered it acceptable.

Our results reconfirmed the psychometric characteristics of the questionnaire observed in other validation studies (14-16). Due to our study, COMPASS-31 became the first cross-culturally adapted self-assessment tool to evaluate dysautonomia in MS patients in Serbia and Croatia. To the best of our knowledge, so far only Italian version of the COMPASS-31 has been validated outside English speaking countries (15).

Clinical validity of COMPASS-31 was assessed by comparing mean values of the domain and total scores with the EDSS score. We found that the EDSS score correlated significantly positively with secretomotor, gastrointestinal, and bladder domains and total COMPASS-31 score. In a recently published study, the correlation between EDSS and COMPASS-31 was not found (8). However, only 41 patients completed the questionnaire, which could have influenced the findings (8). The other possible explanation is that parasympathetic and sympathetic components of the ANS are selectively impaired in different stages of MS (17). While sympathetic cardiovascular dysfunction is associated with clinical activity of the disease, parasympathetic cardiovascular dysfunction generally correlates better with the level of clinical disability as measured by EDSS and disease duration (4,18). In our study, these differences were reflected in the total COMPASS-31 score and its domains among different MS phenotypes. Patients with progressive forms of MS had significantly higher gastrointestinal and bladder domain scores and total COMPASS-31 score as compared with patients with CIS. It has already been reported that bladder and gastrointestinal dysfunctions correlate with higher levels of physical disability and occur more frequently in progressive forms of MS (19,20).

The ROC analysis was used to differentiate between the patients who do and those who do not experience symptoms of orthostatic intolerance. As our study was focused on cardiovascular ANS involvement in MS, a special emphasis was placed on orthostatic intolerance rather than other COMPASS-31 domains.

The cut-off score establishing the level of distinction between patients who do and those who do not experience symptoms of orthostatic intolerance was 13.6. The scale sensitivity was considerably high, indicating the capacity of the scale to identify patients with orthostatic intolerance. Similar relationships were observed for the remaining COMPASS-31 domains.

The main limitation of our study was that no laboratory testing of the ANS was performed to obtain objective neurophysiological evidence of dysautonomia in MS patients. However, we hope that our ongoing project will shed more light on possible discrepancy between subjective and objective ANS dysfunction.

In conclusion, the availability of reliable testing methods remains critical in ANS research. The current validation is part of a large, collaborative, international study on dysautonomia in MS. The present study showed that COMPASS-31 is a valid and acceptable self-assessment instrument for the detection of dysautonomia in MS patients, which may be useful to clinicians and researchers in Serbia and Croatia for detection of this often overlooked clinical problem in MS.
